# Implementation of Patient-Individualized 3D-Printed Models in Undergraduate Students’ Education for Various Prosthetic Treatments: A Cross-Sectional Survey Study

**DOI:** 10.3390/dj12070199

**Published:** 2024-06-27

**Authors:** Andrea Klink, Fabian Engelskirchen, Pablo Kaucher-Fernandez, Fabian Huettig, Ariadne Roehler

**Affiliations:** 1 Department of Prosthodontics, Center for Dentistry, Oral Medicine and Maxillofacial Surgery, University Hospital Tuebingen, 72076 Tübingen, Germany; fabian.engelskirchen@med.uni-tuebingen.de (F.E.); fabian.huettig@med.uni-tuebingen.de (F.H.); 2 Department of Medical Materials Science and Technology, Institute for Biomedical Engineering, University Hospital Tuebingen, 72076 Tübingen, Germany; ariadne.roehler@med.uni-tuebingen.de

**Keywords:** additive manufacturing, skill training, dental education, prosthodontics, CAD/CAM

## Abstract

Background: Due to rapid changes in dental practice, digital technologies have become prominent in undergraduate dental education at German universities in recent years. This shift has prompted a re-evaluation of content as well as teaching methods, particularly in courses where students are prepared for patient treatment. Traditional training on standardized models with resin teeth cannot cover the complexity of individual dental arch configuration encountered in patient situations. This study explores the use of 3D printing technology to create individualized models for prosthetic treatment simulations, aiming to evaluate students’ feedback towards their experience with this training setting. Methods: First, the study describes the design and fabrication of individualized models with exchangeable teeth based on intraoral scans, mounted on connected plates with distance holders that can be fixed to standard phantom heads. Second, students provided feedback through a questionnaire, assessing various aspects such as the effectiveness of the 3D-printed models compared to traditional frasaco models for preparation exercises. Results: The results indicated that the design of the realized models was feasible for preparation training (question no. 4: 93% positive rating) and showed positive perceptions of the 3D-printed models, with students finding them effective for preparation exercises and beneficial in bridging the gap between simulation and real patient situations (question no. 6: 69% positive rating). Conclusions: The study suggests that 3D printing technology offers a valuable tool in dental education, providing realistic and patient-specific scenarios for students to enhance their skills and readiness for clinical practice. Further improvements in material properties in hand with cost-effective approaches are essential for widespread implementation.

## 1. Introduction

In recent years, there have been significant changes in dental education within German dental schools, particularly with the incorporation of digital dentistry technologies into the curriculum to meet the amendment of the federal license regulation (ZApprO) that came into effect in 2021. These changes have prompted revisions in content and teaching methods for both pre-clinical and clinical courses, still aiming to thoroughly prepare students for patient treatments as a practice-ready dentist after graduation [1]. Digital innovations in terms of a “Dentistry 4.0” include technologies such as machine learning, augmented and virtual reality (AR and VR), as well as 3D printing, which are going to become an integral part of modern teaching methods in the long run [2,3]. Today, digital technologies, including intraoral scanning, computer-aided designing (CAD), and in-house production through methods like 3D printing, have become integral to preclinical and clinical training already [4,5].

Nevertheless, acquiring manual skills to handle preparations for direct and indirect restorations is (and will be) still a major topic.

Traditionally, preparation training occurs on simulation units using standardized models in an ideal, healthy, and eugnathic—but this way unrealistic—setting. The teeth in these models are exchangeable and made from a hard plastic material, lacking the realism of tilted, rotated, narrow positioned, elongated, or periodontally damaged teeth with diverging insertion directions [6]. Although these ideal models are commonly used for preparation training just before patient treatment, experiences in clinical courses suggest that preparing teeth in a reduced dentition differ significantly from those of a healthy and full dentition. The scope of clinical prosthodontic treatments in German universities ranges from reduced dentition to be provided with telescopic dentures up to multi-unit fixed dental prostheses (FDP) [7].

Own experiences in these clinical courses showed that the preparation of teeth in a reduced dentition, for example, for double crowns, poses greater challenges for the students compared to three-unit FDPs, for instance. 

Various companies offer models and teeth for simulating endodontic or surgical procedures. In line with the above-mentioned implementation of CAD and additive and subtractive manufacturing into the curriculum, these technologies help to facilitate individual objects for teaching. This way, the necessity of extracted human teeth is lost when it comes to dentition [8]. 

In particular, Peters et al. developed a special 3D-printed tooth replica instead of natural extracted teeth in an attempt to improve students’ skills in root canal treatments. However, they did not find significant improvement and recommended further improvements in resin materials to better simulate natural enamel and dentine properties [9]. Höhne et al. designed 3D-printed teeth with varied layering and material properties to enhance students’ preparation of technique training [10]. In 2020, Hanisch et al. explored the use of individualized 3D-printed surgical training models to simulate apicoectomy based on real patient data. Compared to commercial typodont models, the 3D-printed models provide a more realistic simulation [11,12]. Reymus et al.’s working group developed and evaluated an interdisciplinary single 3D-printed model suitable for root canal treatment, post insertion, and implant insertion. In a survey, students rated the treatments on these models as realistic and comparable to real patient situations [13,14].

The aim of the present study was to simulate the individual prosthetic treatment cases in dental simulation units with phantom heads (KaVo-Kerr Comp., Biberach, Germany) in order to better prepare students for their patient treatments in undergraduate clinical courses. These measures could improve dental education, enhance students’ confidence and skills in complex tooth preparations, and make patient treatment more time efficient. Thus, students can be made aware of potential problems in advance, allowing them to prevent complications during actual patient treatment. Therefore, a workflow should be established that allows for the in-house manufacturing of individual patient case models based on the intraoral scan that can be mounted in a standardized phantom head. These 3D-printed models should contain the removable artificial teeth of comparable hardness to standardized phantom teeth (e.g., #ANKA 4-Z, frasaco GmbH., Tettnang, Germany). 

After preparation trainings with these models, the students should share their experiences and give feedback within a questionnaire regarding feasibility and transferability towards the later treated clinical situation.

Thereby, the study did not follow any hypothesis due to its exploratory character towards feasibility.

## 2. Materials and Methods

### 2.1. Setup of a 3D-Printed Individual Training Model

#### 2.1.1. Mounting Plate for the Phantom Head including Distance Holders

**Figure 1:**
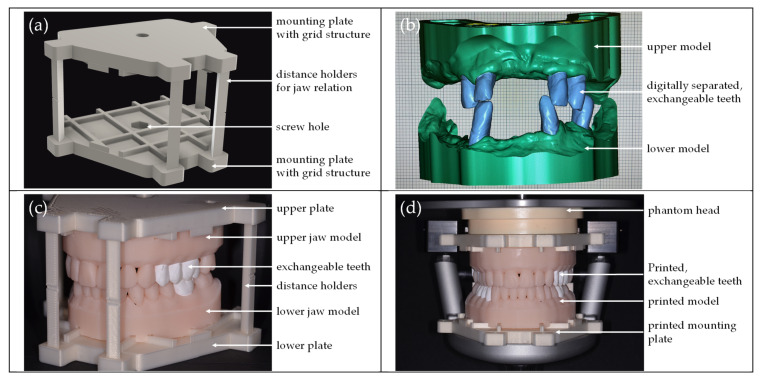
(**a**) Digital design of the mounting parts, upper and lower mounting plates with a grid structure and a screw hole for fastening in the phantom head, four distance holders to simulate jaw relations, and retrieving the occlusion plane. (**b**) Digital design of the models with digitally separated, exchangeable teeth in blue. (**c**) All printed parts mounted together: The upper and lower mounting plates and distance holders were printed using the FFF technique; the upper and lower jaw were printed with the SLA technique. (**d**) All printed parts in the phantom head.

Initially, a plate for mounting the models in a phantom head (#P6/3 Pro, frasaco GmbH, Tettnang, Germany) was designed using construction software (Fusion360, Autodesk Inc., San Rafael, CA, USA). A screw hole was strategically positioned to align with the receptacle in the phantom head, allowing for secure mounting with the screw. The mounting plate, featuring a grid structure on one side, aimed to provide ample support for models to adhere securely. This design was replicated for both upper and lower jaw models. Additionally, the mounting plates included receptacles for four distance holders, simulating the actual position of the upper jaw in relation to the lower jaw (Figure 1a). The 3D printing process for both mounting plates and spacers utilized a material extrusion 3D printer MK3S (Prusa Research a.s., Prague, Czech Republic) with a polylactide (PLA) filament (Prusament PLA Vanilla White, Prusa Research a.s.). The average cost of materials per case for mounting parts was EUR 2.37 (2 mounting plates, 4 spacers).

The mounting plate dataset is provided (in an updated/improved version) by the authors for download as an STL file at https://doi.org/10.5281/zenodo.11174653 (accessed on 23 June 2024).

#### 2.1.2. Jaw Models with Exchangeable Teeth for Preparation

Each student was asked to acquire a digital intraoral scan (IOS) of their patient using a Medit i500 (Medit, Seoul, Republic of Korea). Subsequently, the scans were exported, and the model design was completed by a consistent operator in Ceramill Mind (Amann Girrbach GmbH, Pforzheim, Germany) on the exocad operating system (exocad GmbH, Darmstadt, Germany). The scan data for the upper jaw, lower jaw, and occlusion were imported into the model creator module of the software. Considering the 40 mm distance between the receptacles of the phantom head, the model height was adjusted to 42 mm. To minimize material waste, scans were manually trimmed, retaining essential structures such as the alveolar ridge and teeth. The specific teeth for preparation were separated from the rest of the model. The exchangeable teeth were selected using the FDI tooth numbering system; afterwards, the software was able to detect the correct margin line of most of these teeth. If the margin line was not detected automatically, the line was corrected manually by determining additional points. Parameters for the model dies, such as pin height (1.5 mm), die shape (10°), extrusion of preparation margin (0 mm), horizontal shaft gap (0 mm) or vertical shaft gap (0.2 mm), were fine-tuned during initial trials to ensure good friction. The final step involved uploading the mounting plates to the software as attachments in the correct three-dimensional orientation (plate-to-plate distance 40 mm) and subtracting from the models. This process resulted in the negative form of the grid on the bottom side of the models (Figure 1b).

The model bases and removable teeth were exported in a standard tessellation language (STL) file format. Due to differing requirements for the model base and teeth, they were produced using different materials in the stereolithography (SLA) 3D printer Form 3B (Formlabs Inc., Somerville, MA, USA). The model bases (average cost of materials per case for models: EUR 2.70–3.30 (1 upper jaw model, 1 lower jaw model)) were printed in Model Resin V3 (Formlabs Inc.), while the removable teeth (average cost of materials per case for teeth: EUR 0.30–8.40 (1–8 exchangeable teeth, different type of teeth)) were printed in Rigid 10K Resin V1 (Formlabs Inc.) to closely mimic the feel and preparability of natural teeth. This also allowed students the flexibility to print and replace a tooth for preparation multiple times. Post-processing adhered to manufacturer instructions, involving washing (models for 10 min and teeth two times for 10 min in Form Wash (Formlabs Inc.)) and curing (models for 5 min at 60 °C and teeth for 60 min at 70 °C in Form Cure (Formlabs Inc.)) for the respective materials.

#### 2.1.3. Assembly of the Training Model

Removable teeth were inserted into the model base, and the model base was then affixed to the mounting plate following the prepared grid structure. Distance holders were placed, ensuring that each model base fit into only one position on the mounting plate. Since the IOS included the digital recording of the jaw relation, the spacers’ attachment aimed to ensure the accurate positioning of the occlusion plane (Figure 1c). Finally, the mounting plates with models and removable teeth were secured in the phantom head using screws on each side (Figure 1d). 

### 2.2. Students Preparation and Feedback 

Each 9th semester student, previously trained on conventional frasaco preparation teeth (standard teeth ANA-4 Z, frasaco GmbH, Tettnang, Germany) in preclinical courses, was tasked with practicing preparation on the removable teeth of the individualized 3D-printed models. This practice session required planning and execution according to the associated patient’s treatment plan. Subsequently, each student showcased the preparation on the model to an assistant doctor for evaluation. Upon successful preparation of the individualized 3D-printed teeth, students replicated the same preparation on their respective patients. If the treatment plan had changed between teeth preparation and patient preparation, or if the in vivo situation required a different type of preparation, adjustments were made accordingly. Conventional rotating instruments, such as diamond-coated dental burrs in various shapes (e.g., torpedo, round head bur, bud, etc.), were used for preparations under constant water cooling of 50 mL per minute.

#### Questionnaire

A summary of all questions in the questionnaire is shown in Table 1.

Following preparation on conventional frasaco teeth during preclinical training, 3D-printed teeth, and patients’ teeth, all students were asked to complete a questionnaire assessing the effectiveness of preliminary preparations. Participants received instructions on completing the questionnaire, and each questionnaire was assigned an anonymized pseudonym created by the participants themselves. Pseudonyms comprised initial letters or numbers from categories such as parental names, birthdays, places of birth, and personal characteristics.

The questionnaire consisted of 11 questions. The first three questions sought general information about the performed treatment. The responses to the seven following questions were formulated as a Likert scale with seven increments. An odd number of increments (7) was chosen because a neutral response in the middle of the scale, which neither favors one side nor the other, was considered necessary. This allowed each participant to choose either one clear statement on the poles, a neutral position in the middle, or two gradations in between. The questions should reflect students’ opinions on the preparation of 3D-printed models compared to frasaco models. A free-text comment was optional at the end of the questionnaire.

Discrete responses were entered into a data table for descriptive statistics using JMP software package (15.2, SAS Corp., Cary, NC, USA); the free text feedback was analyzed qualitatively by inductive categorization.

## 3. Results

The study was conducted with one semester cohort of whom all students (n = 27) returned their questionnaires. Two participants treated two patients each, resulting in a total of n = 29 datasets available for evaluation. Participants adhered to the pseudonym creation rules, providing legible entries on the questionnaires. All questionnaires were completed clearly and legibly, with no additional incomplete or excluded datasets.

### Datasets

**Figure 2:**
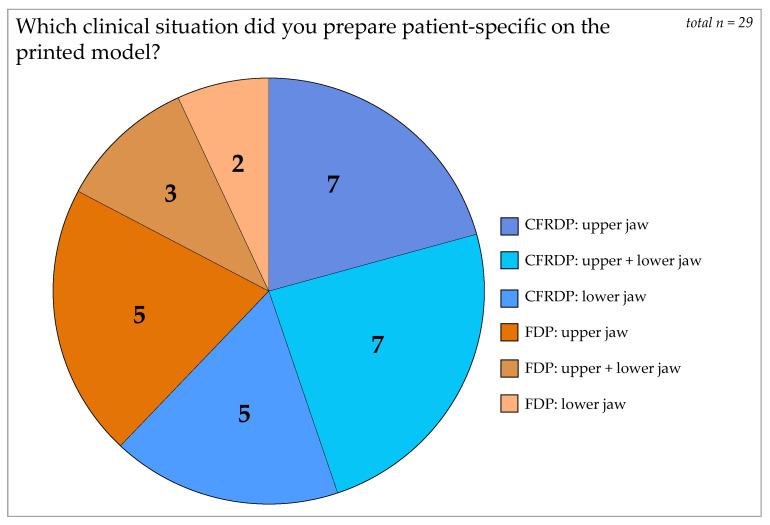
The cake chart on answers to question 1 shows the distribution of clinical situations, which were prepared on the 3D-printed models. Blue shades: different varieties of removable dentures; orange shades: different varieties of fixed dentures.

A total of 11 models for FDPs and 18 models for CFRDP treatments were fabricated, prepared, and evaluated by the participants. For FDP, two were in the lower, five in the upper, and three in both jaws. For CFRDP, five were in the mandible, seven in the maxilla, and seven in both jaws (Figure 2). 

For the second question, more than 29 answers were summarized due to multiple possible responses. In total, 14 students had to remove crowns before treatment, 3 had to remove partial crowns, 9 had to change fillings, and 5 students had to perform a post and core build-up before treatment (Table 2). The final general information about the location of preparation resulted in a total of 67 answers, as multiple responses were possible. In total, 26 students prepared in the dentine, 20 in enamel and 21 in core build-up. 

**Figure 3:**
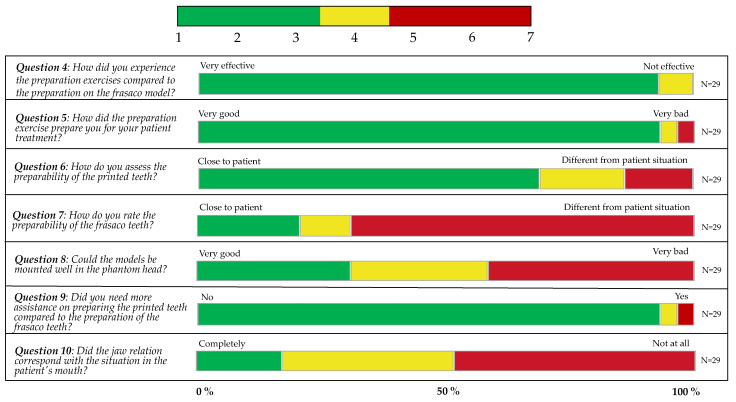
The distribution of all answers per question (4–10), grouped by three parts (green: increment 1–3 on Likert scale; yellow: increment 4; red: increment 5–7), all in percentages.

The distribution of all answers for questions no. 4 to 10 are shown in Figure 3.

The fourth question regarding the perception of the preparation exercise on 3D-printed models compared to the commonly used frasaco models ranged from very effective (increment 1) to increment 4, with a mean of 1.89 ± 0.86. 

The fifth question assessed how well the exercise prepared students for patient treatment, with possible responses ranging from very good (increment 1) to very bad (increment 6), yielding a mean of 2.48 ± 1.02. 

The sixth question inquired about the preparability of 3D-printed teeth, with responses ranging from close to the patient (increment 2) to different from the patient situation (increment 5), resulting in a mean of 3.10 ± 1.05.

Similar to the previous question, the following one addressed the preparability of frasaco teeth, with responses spanning from close to the patient (increment 2) to different from the patient situation (increment 7), and a mean of 4.93 ± 1.36.

The subsequent question aimed to understand how well the models could be assembled in the phantom head. Responses varied from very good (increment 1) to very bad (increment 7), with a mean of 4.14 ± 1.87.

The following question assessed whether participants required more supervision compared to the usual preparation exercises on the frasaco model. Answers ranged from yes (increment 2) to no (increment 7), with a mean of 6.10 ± 1.18.

The final question examined whether the jaw relation of the model corresponded to that in the patient’s mouth. Responses ranged from completely (increment 1) to not at all (increment 7), with a mean of 4.55 ± 1.33. 

All students (n = 27) provided free-text comments. The answers contain four categories: individuality, proximity to the patient, material, and assembly in the phantom head. In summary, students reported feeling better and more confidently prepared for patient treatment thanks to the individual patient models. The students particularly addressed that tilted and elongated teeth could be better prepared, even though they reported to better assess the amount of substance removal and the path of insertion. Regarding material and assembly in the phantom head, the students agreed that the printed teeth were easier to prepare than the frasaco teeth. However, they demanded improvements to the mounting interface to the phantom heads, because some of the models could not be placed correctly on the mounting plates. The same applies for the distance holders that had to be adjusted for a reliable fit.

The identified common opinion highlighted that the efforts involved in the intraoral scan, the production of the individual models, and the practice preparations on the printed models provided worthwhile preparation for clinical patient treatment with increased safety and confidence.

## 4. Discussion

After the initial assembly of the models in the phantom head, it became evident that the correct jaw relation could not always be adequately established. This is also reflected in question no. 10 where a significant number of participants expressed dissatisfaction with the adjustment of the jaw relation or its correspondence with the patient’s actual jaw relation. The distance holders could not be fixed securely in the designated recesses.

Consequently, after immediate oral feedback, the sockets needed a fundamental design adjustment. Instead of placing it on the front face of the mounting plate, it was positioned parallel to the grid structure. Consequently, the spacers no longer had a 90° angulation, enhancing the mechanical stability of the bars; this finding is in line with further fine-tuned printing parameters, which are a reliable fit. Moreover, the grid structure on the mounting plate was minimized to reduce printing time and material costs while maintaining the necessary support for the models. These early oral critiques regarding the support structures for the jaw relation and the mounting of the models on the plates were later echoed in the free-text comments. Another option would be to print the model and the mounting plates in one piece. This would make it easier to adapt the individual structures and could improve the adjustment of the jaw relation. However, such an approach would result in higher printing costs. 

Another crucial point would be the comparison of prior preparations on frasaco models with those on a patient-specific/individualized printed model. The preparations on the frasaco model did not replicate the patient’s situation, but rather mirrored a standardized scenario; this was the same condition for all students. Preparation on the patient-individualized model is reasonable at a later point in time of the curriculum; however, both preparation exercises on the phantom are not completely comparable. 

The objective of using 3D-printed removable teeth was to select a material that closely resembles a natural tooth structure, approaching the clinical situation as closely as possible. Further, the preparation could be repeated without a remake of the complete model due to their exchangeability. This aspect is also addressed in the free texts and is positively evaluated regarding the material choice for the printed teeth.

Responses to questions no. 6 and no. 7, in agreement with the free-text comments, indicated that the preparability of the printed teeth is closer to the patient’s situation than the preparation of frasaco teeth, thus more closely similar to a real situation. Some students even observed that the material properties of the printed teeth were “more comparable to enamel” than the frasaco teeth material. In summary, it can be asserted that the 3D-printed near-patient model represents an improvement in students becoming ready for patient treatment.

Even question no. 9 demonstrated that the need for support from a supervising dentist is less for the 3D-printed models than for previous preparation exercises on frasaco models. However, this can be attributed to the fact that students of the study cohort had already undergone frasaco preparation training during their preclinical courses. It is, therefore, not conclusive if the need for support diminishes through practice, irrespective of the model used. Therewith, it must be highlighted that students should be extensively familiar with their patients’ treatment situation, creating a better foundational prerequisite for the preparation session than a scenario lacking clinical background, enhancing students’ self-confidence and potentially leading to better results.

Nevertheless, the time required to design and fabricate the models, individual teeth, and mounting plates is lacking in our data. Since the students did not handle this process themselves, it is provided by the teaching staff. The goal for upcoming semesters is to simplify the process and delegate a significant portion to the students, which will also train their skills to work with CAD and 3D printing on their own. This starts with assembling the components provided, as printed and supported by online instructions, that shall reduce the time burden for teaching staff.

Cost considerations towards printer and material revealed EUR 6 is needed to fabricate all components for both jaws, excluding the exchangeable teeth. As previously mentioned, there was a call for a material of markedly higher quality and price, resulting in approximately EUR 1 per removable tooth. This renders the total cost for this model higher than for a conventionally printed model without removable teeth, but is more sustainable for multiple iterations of training in this situation, even for other students in advance preclinical training, for instance.

## 5. Conclusions

The integration of 3D printing technology into dental education demonstrates promising results in addressing key challenges of clinical training. The patient-specific 3D-printed dental models bridging the gap between simulated exercises and actual patient scenarios provide students with a more realistic and tailored preparation experience. This was experienced positively, as expressed in the students’ feedback, apart from the previously mentioned shortcomings in assembly and mounting of the models. The results suggest that this approach contributes to improved confidence and skill development.

The study underlines the potential of 3D printing to enhance dental education, offering a valuable tool for individualized and realistic training in a phantom setting. However, ongoing improvements in material properties and cost-effective approaches are crucial for broader implementation. As technology continues to advance, the integration of 3D printing in dental education holds promise for optimizing students’ readiness and competence in delivering high-quality patient care. Future endeavors should focus on refining the 3D printing process, addressing challenges in automatization, and establishing a sustainable and accessible framework for widespread adoption in dental curricula. Further research shall quantify the impact of this approach on students’ performance in clinical treatment. 

## Figures and Tables

**Table 1:** Summary of all questions of the questionnaire including answering options.

No.	Questions
1	Which patient-specific clinical situation did you prepare on the printed model? o combined fixed-removable dental prosthesis (CFRDP): (□ Upper Jaw, □ Lower Jaw) o fixed dental prosthesis (FDP): □ Upper Jaw, □ Lower Jawo o other (multiple answers possible)
2	What procedures did you perform on the teeth before preparation and after initial intraoral scan? o removal of crowns (EKR full) o removal of partial crowns (EKR part) o Change of fillings o post and core build-up o other (multiple answers possible)
3	The preparation of the real tooth was located: o in tooth enamel o in tooth dentin o in core build-up (multiple answers possible)
4	How did you experience the preparation exercises compared to the preparation on the frasaco model? very effective □ - □ - □ - □ - □ - □ - □ not effective
5	How did the preparation exercise prepare you for your patient treatment? very good □ - □ - □ - □ - □ - □ - □ very bad
6	How do you assess the preparability of the printed teeth? close to patient □ - □ - □ - □ - □ - □ - □ different from patient situation
7	How do you rate the preparability of the frasaco teeth? close to patient □ - □ - □ - □ - □ - □ - □ different from patient situation
8	Could the models be mounted well in the phantom head? very good □ - □ - □ - □ - □ - □ - □ very bad
9	Did you need more assistance on preparing the printed teeth compared to the preparation of the frasaco teeth? no □ - □ - □ - □ - □ - □ - □ yes
10	Did the jaw relation correspond with the situation in the patient’s mouth? completely □ - □ - □ - □ - □ - □ - □ not at all
11	Compared with the preparation on the frasaco teeth what did you like better or worse; and why? (freetext)

**Table 2:** Results for question 2—the distribution of what and which pre-treatment procedures were necessary after the intraoral scanning and before preparing the printed teeth. Additionally, results for question 3—the distribution of which structures were included in the preparation of the real patient situation. As more than one structure could be named, the total count is more than the participant count.

**Question 2**: What procedures did you perform on the teeth before preparation and after intraoral scan?	
Removal of crowns	14
Removal of partial crowns	3
Change of fillings	9
Post and core build-up	5
total n = 33	
**Question 3**: The preparation of the real tooth was located:	
Preparation in enamel	20
Preparation in dentin	26
Preparation in core build-up	21
total n = 67	

## Data Availability

The STL files of the mounting plates can be downloaded at: https://doi.org/10.5281/zenodo.11174653 (accessed on 23 June 2024).
